# Survival Trends in Urothelial Cancer Before and After ICIs and Antibody Drug Conjugates

**DOI:** 10.1001/jamanetworkopen.2025.19524

**Published:** 2025-07-09

**Authors:** Yi Lian, Teja Voruganti, Jenny Lu, Qi Long, Ronac Mamtani

**Affiliations:** 1 Department of Biostatistics, Epidemiology, and Informatics, University of Pennsylvania, Philadelphia; 2 Division of Hematology and Oncology, Department of Medicine, University of Pennsylvania, Philadelphia; 3 Perelman School of Medicine at the University of Pennsylvania, Philadelphia; 4 Abramson Cancer Center, University of Pennsylvania, Philadelphia

## Abstract

This cohort study compares population-level temporal trends in survival before and after using immune checkpoint inhibitors and antibody-drug conjugates among patients with metastatic urothelial cancer.

## Introduction

Patients with metastatic urothelial cancer (MUC) historically have poor long-term survival (ie, less than a 5% chance of 5-year survival).^1^ The introduction of novel therapies, such as immune checkpoint inhibitors (ICIs) and antibody-drug conjugates (ADCs), have prolonged survival in clinical trial settings.^2,3,4^ However, it is unclear how outcomes have changed in clinical practice. We compared population-level temporal trends in survival before and after the introduction of ICIs (ie, pembrolizumab or atezolizumab) and ADCs (ie, enfortumab vedotin) among patients with MUC receiving routine care.

## Methods

We conducted a retrospective cohort study of patients with MUC who initiated first-line systemic therapy between January 1, 2011, and September 30, 2023, with follow-up through September 30, 2024. We used data from the US nationwide electronic health record (EHR)-derived Flatiron Health database.^5^ Untreated patients or those receiving clinical trial drugs were excluded. The study sample was divided into 3 time periods: before ICI approval (2011 to 2016), after ICI approval but before ADC approval (2017 to 2019), and after ADC approval (2020 to 2023). Inverse probability of treatment weighting (IPTW)–adjusted Kaplan-Meier curves compared temporal trends in median overall survival (OS) and 3-year OS across 3 cohorts of patients defined by time period and adjusted for patient, tumor, and practice factors. Follow-up continued until death or last structured activity in the EHR prior to date of data extraction (September 30, 2024). The University of Pennsylvania institutional review board approved the study with a waiver of informed consent because deidentified retrospective data were used. This cohort study follows the Strengthening the Reporting of Observational Studies in Epidemiology (STROBE) reporting guideline.

## Results

We summarized the baseline characteristics of our sample in the Table, which were included in the IPTW model and were well balanced. Our sample included 8955 patients (mean [SD] age, 72.07 [9.50] years) initiating first-line therapy for MUC (2576 [28.8%] before ICI approval, 2943 [32.9%] after ICI approval but before ADC approval, and 3436 [38.4%] after ADC approval). IPTW-adjusted Kaplan-Meier survival estimates are shown in the Figure. We reported 95% bootstrap CIs and associated *P* values using 10 000 resamples (with replacement), retaining IPTW. From 2011 to 2023, the adjusted 3-year survival probability increased from 19.2% (95% CI, 17.5%-20.9%) before ICI approval to 23.3% (95% CI, 21.5%-24.8%) after ICI approval but before ADC approval to 27.6% (95% CI, 25.7%-29.4%) after ADC approval (*P* < .001). Similarly, median survival of treatment initiators increased from 11.1 (95% CI, 10.7-11.9) months in the 2011 to 2016 group before ICI approval to 13.6 months (95%, 12.7-14.7) in the 2020 to 2023 group after ICI and ADC approval (*P* < .001).

**Table.** Baseline Characteristics by Time Period Before and After Inverse Probability Weighting^a^

Characteristic	Unweighted population				Weighted population			
	Patients, No. (%)			SMD	Patients, No. (%)			SMD
	Before ICI	After ICI before ADC	After ADC		Before ICI	After ICI before ADC	After ADC	
No.	2576	2943	3436	NA	2945	2994	3015	NA
Age, mean (SD), y	70.84 (9.65)	72.30 (9.42)	72.78 (9.36)	0.205	72.21 (9.21)	72.23 (9.25)	72.27 (9.31)	0.005
Birth sex								
Female	672 (26.1)	763 (25.9)	965 (28.1)	0.022	782 (26.6)	788 (26.3)	801 (26.6)	0.003
Male	1904 (73.9)	2180 (74.1)	2471 (71.9)		2163 (73.4)	2205 (73.7)	2214 (73.4)	
Race								
Black	131 (5.1)	147 (5.0)	161 (4.7)	0.025	141 (4.8)	145 (4.9)	135 (4.5)	0.003
Other race	350 (13.6)	458 (15.6)	430 (12.5)		413 (14.0)	421 (14.1)	383 (12.7)	
White	2095 (81.3)	2338 (79.4)	2845 (82.8)		2390 (81.2)	2426 (81.0)	2496 (82.8)	
Stage at diagnosis								
0	14 (0.5)	14 (0.5)	15 (0.4)	0.208	10 (0.4)	12 (0.4)	13 (0.4)	0.013
1	37 (1.4)	46 (1.6)	74 (2.2)		42 (1.4)	47 (1.6)	56 (1.9)	
2	137 (5.3)	321 (10.9)	528 (15.4)		226 (7.7)	320 (10.7)	399 (13.2)	
3	144 (5.6)	335 (11.4)	654 (19.0)		253 (8.6)	352 (11.8)	464 (15.4)	
4	2244 (87.1)	2227 (75.7)	2165 (63.0)		2411 (81.9)	2260 (75.5)	2081 (69.0)	
ECOG PS								
<2	2342 (90.9)	2486 (84.5)	2947 (85.8)	0.029	2666 (90.5)	2573 (85.9)	2573 (85.3)	0.001
≥2	234 (9.1)	457 (15.5)	489 (14.2)		279 (9.5)	420 (14.1)	442 (14.7)	
Smoking history								
No	713 (27.7)	776 (26.4)	945 (27.5)	0.013	787 (26.7)	804 (26.9)	809 (26.9)	0.002
Yes	1863 (72.3)	2167 (73.6)	2491 (72.5)		2157 (73.3)	2189 (73.1)	2205 (73.1)	
Location								
Midwest	336 (13.0)	447 (15.2)	385 (11.2)	0.037	387 (13.2)	408 (13.6)	374 (12.4)	0.003
Northeast	380 (14.8)	399 (13.6)	468 (13.6)		405 (13.8)	420 (14.0)	417 (13.9)	
South	1492 (57.9)	1688 (57.4)	2115 (61.6)		1752 (59.5)	1743 (58.2)	1797 (59.6)	
West	368 (14.3)	409 (13.9)	468 (13.6)		400 (13.6)	421 (14.1)	425 (14.1)	
Insurance								
Commercial	1575 (61.1)	1741 (59.2)	2100 (61.1)	0.041	1816 (61.7)	1815 (60.6)	1803 (59.8)	0.003
Government	825 (32.0)	904 (30.7)	1024 (29.8)		907 (30.8)	919 (30.7)	940 (31.2)	
Other	176 (6.8)	298 (10.1)	312 (9.1)		221 (7.5)	259 (8.7)	270 (9.0)	
Practice								
Academic	442 (17.2)	582 (19.8)	760 (22.1)	0.050	569 (19.3)	584 (19.5)	602 (20.0)	0.006
Community	2134 (82.8)	2361 (80.2)	2676 (77.9)		2376 (80.7)	2409 (80.5)	2413 (80.0)	
Primary site								
Bladder	2013 (78.1)	2286 (77.7)	2628 (76.5)	0.022	2293 (77.9)	2330 (77.8)	2335 (77.5)	0.004
Renal pelvis	339 (13.2)	369 (12.5)	452 (13.2)		380 (12.9)	385 (12.9)	390 (13.0)	
Ureter	190 (7.4)	270 (9.2)	330 (9.6)		250 (8.5)	258 (8.6)	265 (8.8)	
Urethra	34 (1.3)	18 (0.6)	26 (0.8)		21 (0.7)	20 (0.7)	23 (0.8)	

Abbreviations: ADC, antibody-drug conjugate therapy; ECOG PS, Eastern Cooperative Oncology Group Performance Status; ICI, immune checkpoint inhibitor therapy; NA, not applicable; SMD, standardized mean difference.

^a^ Baseline characteristics by time period of treatment initiation in the original study population (unweighted) and the inverse probability of treatment weighting (IPTW)–adjusted study population. To balance systematic differences in covariates across the 3 cohorts, multinomial generalized boosted models estimated the propensity scores using the 10 covariates listed in the Table. Multiple imputation was used to impute missing values.

**Figure.**
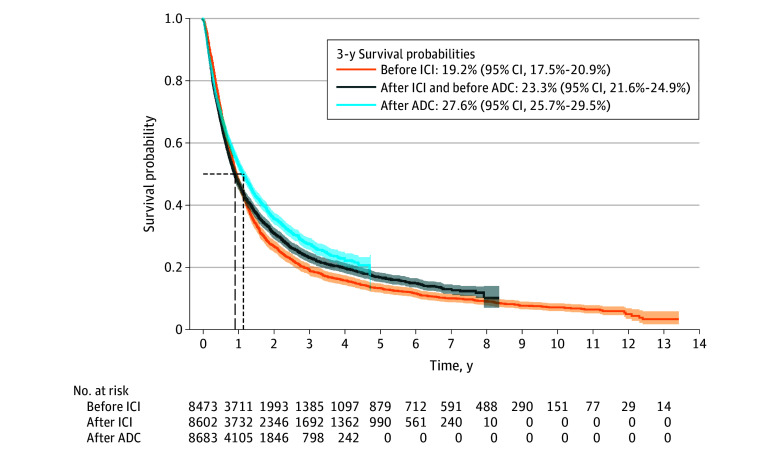
Cumulative Survival Probabilities by Time Period of Treatment Initiation The Kaplan-Meier survival curves and 3-year survival estimates are inverse probability weighted based on the propensity score model fitted using the 10 covariates listed in the Table. ADC indicates antibody drug conjugate therapy; ICI, immune checkpoint inhibitor therapy.

## Discussion

 In this study, we found population-level increased in survival after the introduction of novel cancer therapeutics (ie, ICIs and ADCs). This difference reflects a clinically meaningful 8.4 percentage point absolute improvement and 44% relative improvement in long-term survival (3-year OS, 19.2% to 27.6%) for patients initiating therapy in contemporary practice. Because approximately 38.1% of patients initiated an ICI or ADC by September 30, 2024, our results suggest that implementation of newer therapies was likely associated with the clinical benefit observed. Only 1 prior study examined temporal trends in survival among patients with MUC, but the analysis limited cohort entry to 2020 and was therefore unable to assess survival changes after ADC adoption in 2020.^6^ Our data allow clinicians and researchers to characterize contemporary survival benefits of ICIs and ADCs for patient in routine care and may be used to benchmark outcomes from future clinical trials. Limitations of this analysis include patient-level confounding, which was reduced by weighted comparisons across time periods; limited follow-up after approval of combination ICI and ADC (ie, enfortumab and pembrolizumab) in December 2023, which could underestimate survival benefit; and limited generalizability of results to academic practices because Flatiron data are predominantly community-based.

## Notes

Supplement.
Data Sharing Statement
