# Supplementary material for: Survival Trends in Urothelial Cancer Before and After ICIs and Antibody Drug Conjugates
Source: JAMA Netw Open. 2025 Jul 9;8(7):e2519524. doi: 10.1001/jamanetworkopen.2025.19524 (PMC12242697; doi:10.1001/jamanetworkopen.2025.19524)
Supplement: Supplement. — Data Sharing Statement [file jamanetwopen-e2519524-s001.pdf]

Data Sharing Statement

Data
Data available: No

Additional Information
Explanation for why data not available: The data that support the findings of this study have been originated by Flatiron Health, Inc. These de-identified data may be made available upon request and are subject to a license agreement with Flatiron Health; interested researchers should contact DataAccess@flatiron.com to determine licensing terms.
